# CyTOF profiling identifies location-specific peripheral immune checkpoint and immune cell subset in mild ischemic stroke

**DOI:** 10.3389/fimmu.2026.1739324

**Published:** 2026-01-27

**Authors:** Hang Hang, Yang Yao, Likun Wang, Cuiying Liu, Jing Zhao, Baohui Xu, Heng Zhao, Guofeng Wu

**Affiliations:** 1 Emergency Department, The Affiliated Hospital of Guizhou Medical University, Guiyang, Guizhou, China; 2 Beijing Institute of Brain Disorders, Laboratory of Brain Disorders, Ministry of Science and Technology, Joint Innovation Center for Brain Disorders, Capital Medical University, Beijing, China; 3 Department of Neurology, Tianjin Medical University General Hospital, Tianjin, China; 4 Department of Neurology, Neurosurgery, and Ophthalmology, Tongren People’s Hospital, Tongren, Guizhou, China; 5 School of Nursing, Capital Medical University, Beijing, China; 6 Division of Vascular Surgery, Department of Surgery, Stanford University, Stanford, CA, United States

**Keywords:** cortical stroke, immune checkpoints, mass cytometry, mild ischemic stroke, subcortical stroke

## Abstract

**Introduction:**

Mild ischemic stroke accounts for over half of all stroke cases, yet how peripheral immune responses evolve over time—and how they differ by infarct location—remains poorly defined.

**Methods:**

Peripheral blood was collected from ten patients with mild ischemic stroke and five matched controls at days 1, 3, and 7 after onset. Patients were stratified by cortical or subcortical infarction. High-dimensional mass cytometry was used to characterize immune cell composition and immune checkpoint expression.

**Results:**

Subcortical infarction was associated with sustained expansion of classical monocytes, persistent reduction of intermediate monocytes, and delayed PD-1/PD-L1 regulatory signaling, indicating prolonged myeloid-driven inflammation. In contrast, cortical infarction exhibited a more balanced monocyte profile and earlier PD-1 upregulation on dendritic cells and classical monocytes. CD4⁺ and CD8⁺ T-cell subsets showed distinct, location-dependent dynamics: cortical infarction induced earlier modulation of memory and regulatory phenotypes, whereas subcortical infarction produced slower but more persistent shifts. CCR5-defined CD8⁺ T-cell subsets also differed markedly, with subcortical infarction showing enrichment of CCR5⁺ effector cells, reduced checkpoint expression, and contraction of the CCR5⁻ compartment.

**Discussion:**

Peripheral immune remodeling in mild ischemic stroke displays clear infarct location–specific trajectories. These findings highlight infarct topology as a critical determinant of post-stroke immune regulation and support the development of location-adapted immunomodulatory strategies.

## 1 Introduction

Ischemic stroke is a focal cerebrovascular event and a systemic immunological challenge that progresses through two overlapping phases: acute neuroinflammation followed by peripheral immunosuppression (1). Among peripheral immune responders, monocytes are rapidly recruited to the ischemic brain and differentiate into macrophages with either pro-inflammatory (M1-like) or anti-inflammatory/reparative (M2-like) functions (2). T cells also exert diverse effects after ischemic stroke. CD4^+^ helper T (Th) cells can polarize toward Th1 or Th2 subsets, with Th1 cells amplifying neuronal injury through interferon-γ, while Th2 cells secrete cytokines such as IL-4 that support neuroprotection (3). Regulatory T cells (Tregs) suppress excessive inflammation and facilitate recovery (4). CD8^+^ cytotoxic T cells damage neurons via perforin and granzyme release but may subsequently shift toward regulatory or exhaustion phenotypes that limit inflammation (5). Systemic immunosuppression typically follows initial immune activation, characterized by lymphopenia, T cell exhaustion, and monocyte deactivation (6). This state predisposes patients to infections, particularly pneumonia and sepsis, which contribute substantially to post-stroke morbidity and mortality (7). Although involving autonomic nervous system activation and immune checkpoint signaling, the mechanisms driving this biphasic response remain incompletely understood (8, 9).

Immune checkpoints are increasingly recognized as key regulators of post-stroke immunity. Programmed cell death-1 (PD-1) and its ligand PD-L1 are upregulated after stroke and exert context-dependent effects (10, 11). PD-1 engagement can restrain overactive T cells and reduce collateral damage (12), but excessive signaling may promote stroke-induced immunosuppression and infection risk (13, 14). Other inhibitory receptors, including Tim-3, T cell immunoreceptor with Ig and ITIM domains (TIGIT, and VISTA, are well known to dampen T cell activity in cancer and chronic infection (15–17). Their roles in stroke are less defined (16, 18), but they may contribute to immune exhaustion and deserve further study. Beyond serving as markers of immune status, these molecules represent potential therapeutic targets. Preclinical studies suggest that manipulating checkpoint pathways influences stroke outcomes: blocking PD-L1 reduced neuroinflammation in experimental models (19), whereas PD-1 engagement can limit excessive immune activation (11). These findings point to checkpoint modulation as a possible strategy to balance protective and harmful immunity.

Despite advances in understanding immune responses after ischemic stroke, the spatiotemporal dynamics of post-stroke immunity remain poorly characterized. Most studies have been cross-sectional or restricted to a few markers, missing the evolving nature of immune responses. High-dimensional techniques such as mass cytometry (CyTOF) allow single-cell resolution profiling of immune subsets and checkpoint expression, but longitudinal studies in patients remain scarce.

Infarct location is another determinant of systemic immunity that has received little attention. Compared to subcortical infarcts, cortical infarcts-particularly those involving autonomic regions such as the insula-are associated with stronger inflammation and more pronounced immunosuppression (20–22). These differences likely reflect both the extent of tissue damage and differential brain–immune signaling. However, direct comparisons of immune responses between cortical and subcortical infarcts are limited.

Therefore, ischemic stroke induces location-specific and time-dependent remodeling of peripheral immunity, characterized by changes in monocyte and T cell subsets and their checkpoint expression. To test this, CyTOF was applied to profile peripheral blood immune cells in patients with mild ischemic stroke-distinguishing cortical from subcortical infarcts-on days 1, 3, and 7 after onset, focusing on immune subset dynamics and checkpoint regulation.

## 2 Materials and methods

### 2.1 Subjects and ethics statement

This study had been approved by the Ethics Committee of Guizhou Medical University (Approval No. 2023228K). Patients were consecutively recruited from the Emergency Neurology Department of the Affiliated Hospital of Guizhou Medical University between December 2023 and April 2024. The study cohort consisted of 10 patients with acute mild ischemic stroke and 5 age- and gender-matched healthy controls.

Inclusion criteria for ischemic stroke patients were: (i) age >18 years; (ii) acute onset of focal neurological deficits consistent with ischemic stroke; (iii) measurable neurological deficits (NIHSS ≤5) (23); (iv) anterior circulation ischemic stroke confirmed by magnetic resonance angiography (MRA) and diffusion-weighted imaging (DWI); and (v) symptom onset between 4.5 and 24 hours before admission. Exclusion criteria included: (i) hemorrhagic stroke; (ii) other central nervous system disorders; (iii) pre-existing neurological dysfunction (mRS >2); (iv) dysphagia; (v) history of chronic arrhythmias or atrioventricular block; (vi) use of antineoplastic, immunosuppressive, or immunomodulatory treatments; and (vii) presence of macular edema.

Controls were approximately 60 years old, had no history of ischemic or hemorrhagic stroke, and were recruited as a cross-sectional reference group, with each individual contributing a single baseline blood sample. The exclusion criteria for controls matched those applied to the ischemic stroke group.

All patients underwent diffusion-weighted MRI (DWI) within 24 hours of admission. Two experienced ischemic stroke neurologists independently reviewed all scans and classified infarct location. Cortical infarction was defined as ischemic lesions involving the cerebral cortex on DWI. Subcortical infarction was defined as lesions confined to deep gray- or white-matter structures, including the basal ganglia, internal capsule, thalamus, or corona radiata, without extension into the cortical ribbon (24). Disagreements were resolved by consensus. Peripheral blood samples were collected at 24 h, 72 h, and 7 d after admission. Day 1 represents the immediate innate immune activation phase, day 3 corresponds to the secondary inflammatory wave and early lymphocyte redistribution, and day 7 reflects the transition toward early immune resolution, as established in prior studies examining systemic post-stroke immune dynamics. These timepoints align with well-described peripheral immune activation phases following ischemic injury (1, 25). This was an exploratory study, and no formal sample size calculation was performed. A schematic overview of the experimental workflow is provided in **Supplementary Figure 1**.

### 2.2 Single-cell mass cytometry

A mass cytometry panel of 42 metal-conjugated antibodies targeting cell surface markers was used to identify immune phenotypes and functional states (**Supplementary Table 1**). Each lineage was further divided into functional sub-clusters according to differentiation markers, chemokine receptors, and immune checkpoint molecules, as detailed in **Supplementary Table 2**. CyTOF analysis was performed by PLTTech Inc. (Hangzhou, China) following established protocols (26).

Briefly, PBMCs were isolated using Ficoll density-gradient centrifugation, and red blood cells were removed with ACK lysis buffer (Sigma-Aldrich, St. Louis, MI, USA) if necessary. Cells were cryopreserved and thawed using standardized procedures to ensure adequate post-thaw quality, and all samples were stained simultaneously. Approximately 400,000 cells per sample were acquired.

Cells were stained with cisplatin to exclude dead cells, blocked, and incubated with a surface antibody cocktail for 30 min at room temperature. After washing, cells were fixed, permeabilized, and stained with an intracellular antibody panel. A DNA intercalator was added for overnight staining at 4°C. Barcoding was performed, and cells were washed, resuspended in deionized water with 20% EQ beads (Fluidigm, CA, USA), and acquired on a Helios mass cytometer (Fluidigm, CA, USA).

### 2.3 CyTOF data analysis

Mass cytometry does not require fluorescence spillover compensation because metal isotope signals do not overlap; therefore, no compensation was applied. Raw FCS files were normalized using EQ™ Four-Element calibration beads, followed by signal drift correction and batch alignment with the CATALYST workflow (27), including channel range adjustment, warping, and anchor-based batch correction. Immune-cell frequencies are reported as relative proportions within the CD45^+^ population, as absolute cell counts could not be determined due to cell loss during processing and the absence of counting beads (28).

After normalization, samples were debarcoded using a dual-layer barcoding strategy. Data were processed in FlowJo (BD Biosciences, USA), with exclusion of beads (140Ce^-^), debris (191Ir^+^/193Ir^+^), dead cells (194Pt^-^), and doublets (event length < 20). Single, live CD45^+^ leukocytes were retained for downstream analysis. Neutrophils were underrepresented due to CD45^dim expression and loss during Ficoll isolation, whereas basophils remained detectable within the CD45^+^ gate.

Marker expression values were transformed using an arcsinh transformation (cofactor = 5) to stabilize variance and compress high-intensity signals (29). All reported marker intensities represent arcsinh-transformed values. Cell clustering was performed using the X-shift algorithm, with clusters annotated based on canonical marker expression. Data visualization included heatmaps, t-SNE plots, line graphs for temporal dynamics, and bar graphs for group comparisons.

### 2.4 Statistical analysis

CyTOF-derived cell frequencies and arcsinh-transformed marker expression values (cofactor = 5) were analyzed using statistical methods appropriate for the distribution of each variable. Data are presented as mean ± SEM or median with interquartile range (IQR), as appropriate.

Although samples at days 1, 3, and 7 were obtained from the same stroke patients, formal longitudinal inferential modeling was not performed due to the limited sample size and the high dimensionality and distributional characteristics of mass cytometry data. Accordingly, group comparisons were conducted independently at each time point, and temporal patterns within each infarct-location group were summarized descriptively.

At each time point, differences among control, cortical infarction, and subcortical infarction groups were assessed using one-way analysis of variance (ANOVA) with Student–Newman–Keuls *post hoc* testing for normally distributed variables, or the Kruskal–Wallis test with Dunn’s correction for non-normally distributed variables. Two-group comparisons were performed using Student’s *t* test or the Mann–Whitney *U* test, as appropriate.

The control group was sampled in a cross-sectional manner and served as a baseline reference; no time-dependent or within-subject statistical analyses were performed for controls. All statistical analyses were performed using GraphPad Prism (GraphPad Software, San Diego, CA, USA), and a two-tailed *P* value < 0.05 was considered statistically significant.

## 3 Results

### 3.1 Study cohort: demographic and clinical characteristics of patients with mild ischemic stroke

Ten patients with mild ischemic stroke were enrolled and stratified into cortical (n = 5) and subcortical (n = 5) infarction groups based on MRI findings; five matched individuals without ischemic stroke served as controls. The median age was 56 years (IQR: 55-74) in the cortical group and 58 years (IQR: 58-76) in the subcortical group, with 80% males in both groups. Active smoking was reported in 80% of patients, while diabetes was present in one patient per group. Hypertension was more frequent in cortical infarction (80%) than in subcortical infarction (40%).

Baseline parameters showed higher systolic/diastolic blood pressure in ischemic stroke patients (cortical: 154/84 mmHg; subcortical: 160/97 mmHg) compared with controls (127/79 mmHg), whereas admission glucose was lower (cortical: 5.33 mmol/L; subcortical: 4.54 mmol/L vs. controls: 6.61 mmol/L). Median NIHSS was 4 (IQR: 4-5) in cortical and 5 (IQR: 3-5) in subcortical infarction. Laboratory findings included modestly elevated CRP, monocyte counts, and monocyte-to-HDL ratios in both groups relative to controls (**Table 1**).

**Table 1:** Demographic and clinical characteristics of the study population.

Characteristics	Control	Cortical	Subcortical
Age	58 (57-62)	56.0 (55.0-74.0)	58.0 (58.0-76.0)
Sex			
Male	3 (60%)	4 (80.0%)	4 (80.0%)
Female	2 (40%)	1 (20.0%)	1 (20.0%)
Co-morbidites			
Smoking			
Smoker	0/5 (0%)	4/5 (80.0%)	4/5 (80.0%)
Ex-smoker	4/5 (80%)	1/5 (20%)	1/5 (20%)
Non-smoker	1/5 (20%)	0/5(0%)	0/5 (0%)
Diabetes	0/5 (0%)	1/5 (20%)	1/5 (20%)
Coronary artery disease	0/5 (0%)	0/5 (0%)	0/5 (0%)
Hypertension	1/5 (20%)	4/5 (80.0%)	2/5 (40.0%)
Hyperlipidaemia	0/5 (0%)	0/5 (0%)	0/5 (0%)
Previous stroke/ Transient ischemic attack	0/5 (0%)	0/5 (0%)	0/5 (0%)
Family history of stroke	0/5 (0%)	0/5 (0%)	0/5 (0%)
Admission parameters			
First systolic blood pressure(mmHg)	127(113-137)	154.00 (148.00-164.00)	160.00 (152.00-163.00)
First diastolic blood pressure(mmHg)	79(68-80.5)	84.00 (78.00-101.00)	97.00 (91.00-100.00)
Blood glucose on admission	6.61(5.49-8.25)	5.33 (5.33-6.88)	4.54 (3.97-5.24)
National Institutes of Health Stroke Scale	0	4 (4.0-5.0)	5 (3.0-5.0)
Thrombolysis	0/5(0%)	0/5 (0%)	0/5 (0%)
MRI lesion location			
Cortical	–	5/5 (50%)	0/5 (50%)
Subcortical	–	0/5 (50%)	5/5 (50%)
Clinical laboratory examination			
C-reactive protein	–	1.86 (1.49-5.70)	1.68 (1.52-1.83)
White blood cells	7.49(5.5-9.15)	9.40 (7.00-9.70)	6.90 (6.80-7.30)
Lymphocytes	2.24(1.24-1.66)	1.99 (1.92-2.03)	1.87 (1.74-2.34)
Neutrophils	4.82(3.31-5.71)	6.75 (4.38-7.07)	4.30 (4.20-4.51)
Monocytes	0.28(0.25-0.50)	0.57 (0.53-0.62)	0.62 (0.48-0.62)
High density lipoprotein cholesterol	–	0.91 (0.86-1.29)	0.91 (0.87-0.93)
Homocysteine	–	15.00 (12.70-16.50)	23.40 (12.80-23.50)
Monocyte-high density lipoprotein cholesterol ratio	–	0.65 (0.44-0.68)	0.55 (0.53-0.67)

### 3.2 High-dimensional cytometric analysis of peripheral blood in the acute phase of mild ischemic stroke

**Figure 1:**
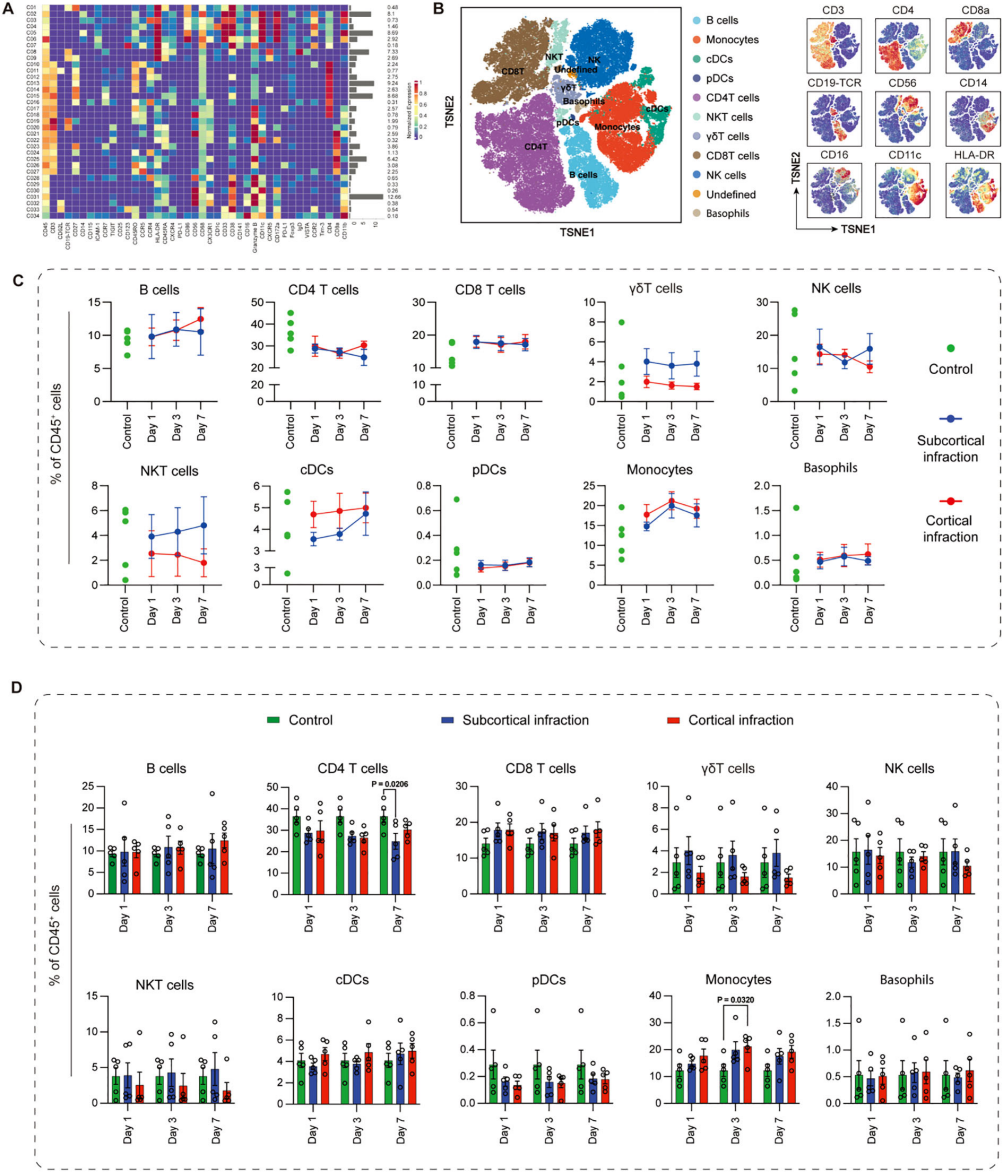
Immune cell population dynamics in ischemic stroke patients with cortical and subcortical infarctions. **(A)** CD45^+^ PBMCs were clustered into 34 phenotypic groups and merged into 11 major immune subsets based on canonical markers: B cells (CD19^+^), CD4^+^ T cells (CD3^+^CD4^+^), CD8^+^ T cells (CD3^+^CD8^+^), γδT cells (TCRγδ^+^), NK cells (CD56^+^CD3^-^), NKT cells (CD56^+^CD3^+^), classical monocytes (CD14^+^CD16^-^), intermediate monocytes (CD14^+^CD16^+^), non-classical monocytes (CD14^-^CD16^+^), cDCs (CD11c^+^HLA-DR^+^), pDCs (CD123^+^HLA-DR^+^), and basophils (CD123^+^CD11c^-^). **(B)** t-SNE visualization of the 11 subsets. **(C)** Subset frequencies at days 1, 3, and 7 in cortical (red) and subcortical (blue) infarction groups. Green dots indicate individual values from cross-sectionally sampled control subjects and are shown as baseline references. **(D)** Group comparisons of immune-subset frequencies. Green bars represent the same cross-sectionally sampled control subjects and are shown as baseline references. All expression values are arcsinh-transformed (cofactor = 5). Data are shown as mean ± SEM. Statistical significance is shown as exact P values within the figure.

To profile systemic immune alterations during the acute phase of mild ischemic stroke, we performed high-dimensional mass cytometry (CyTOF) on PBMCs from ischemic stroke patients and controls. A total of 34 immune clusters were identified within CD45^+^ PBMCs and subsequently grouped into 11 major subsets, including B cells, CD4^+^ T cells, CD8^+^ T cells, γδT cells, natural killer (NK) cells, NKT cells, monocytes, dendritic cells (conventional cDCs and plasmacytoid pDCs), basophils, and undefined populations (**Figure 1A**).

t-SNE analysis demonstrated clear annotation and separation of these subsets based on surface marker expression (**Figure 1B**). Longitudinal assessment revealed modest but measurable variations between cortical and subcortical infarction groups across days 1, 3, and 7 (**Figure 1C**). Notably, CD4^+^ T cell frequencies showed a transient reduction in the subcortical group at day 7 (P = 0.021). In contrast, monocyte frequencies were significantly increased in the cortical infarction group compared with controls (P = 0.032), indicating location-specific differences in early myeloid responses. The frequencies of NK cells, as well as other immune cell populations assessed, remained stable across groups and time points, without significant changes (**Figure 1D**).

### 3.3 Alterations in myeloid cell subsets and immune checkpoints during acute mild ischemic stroke

**Figure 2:**
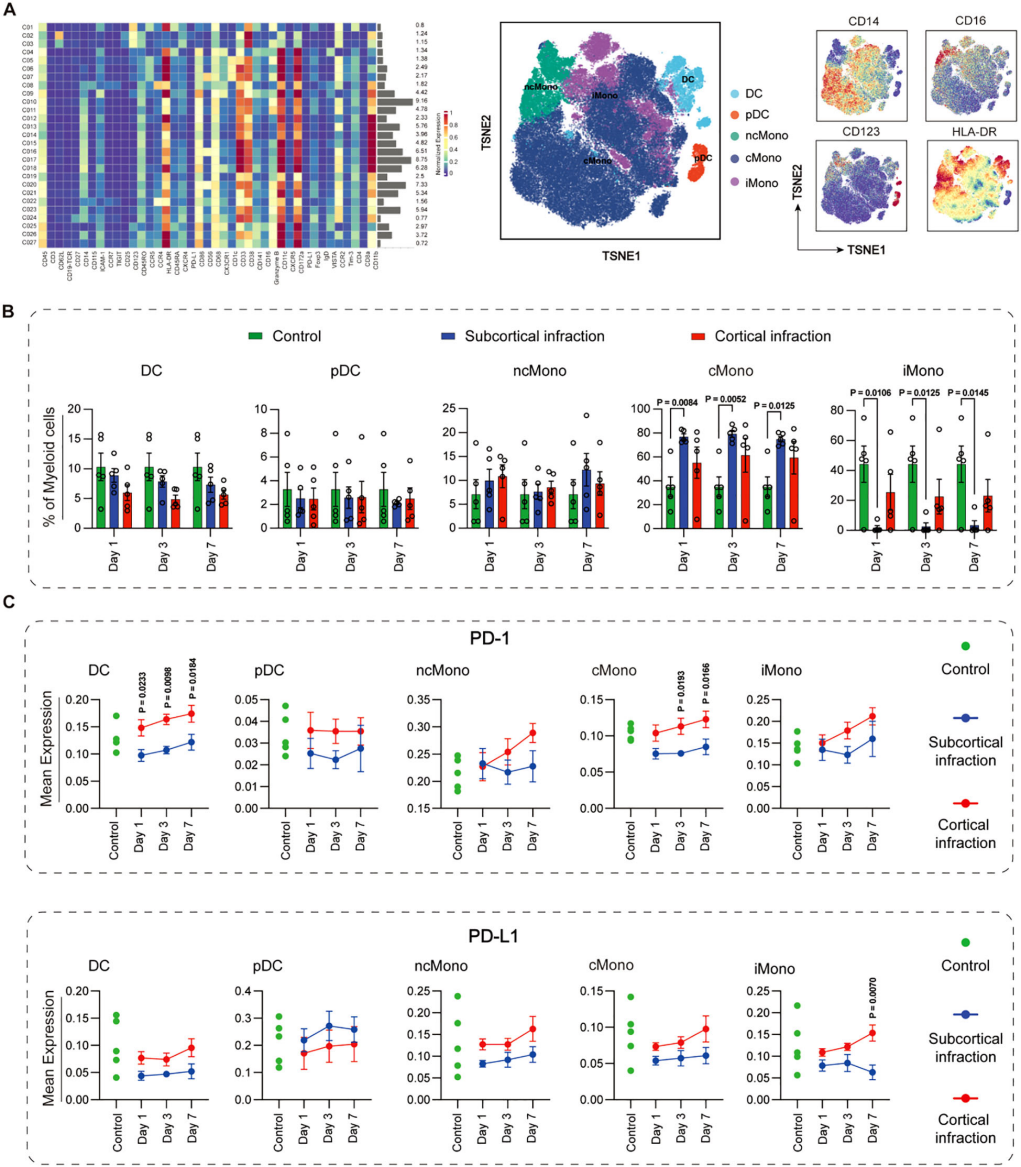
Temporal dynamics and immune checkpoint expression of myeloid cell subsets in cortical and subcortical infarction groups. **(A)** Myeloid cells were clustered and annotated using canonical markers into classical monocytes (CD14^+^CD16^-^), intermediate monocytes (CD14^+^CD16^+^), non-classical monocytes (CD14^-^CD16^+^), cDCs (CD11c^+^HLA-DR^+^), and pDCs (CD123^+^HLA-DR^+^). **(B)** Frequencies of myeloid subsets in cortical (red) and subcortical (blue) infarction groups across days 1, 3, and 7. Green bars represent cross-sectionally sampled control subjects and are shown as baseline reference values. **(C)** Expression of PD-L1 and PD-1 across myeloid subsets. Green dots represent individual values from the same cross-sectionally sampled control subjects shown in panel B and are presented as baseline reference values. All marker expression values are arcsinh-transformed (cofactor = 5). Data represent mean ± SEM. Statistical significance is shown as exact P values within the figure.

Myeloid cells were further classified into five subsets: classical monocytes (cMono), intermediate monocytes (iMono), non-classical monocytes (ncMono), conventional dendritic cells (cDCs), and plasmacytoid dendritic cells (pDCs) (**Figure 2A**). Across all time points, cMono frequencies were significantly higher in the subcortical infarction group compared with controls (day 1: P = 0.008; day 3: P = 0.005; day 7: P = 0.001). Cortical infarction patients also exhibited increased cMono levels relative to controls, although these increases did not reach statistical significance and did not differ from those observed in the subcortical group. Conversely, iMono frequencies were consistently reduced in the subcortical infarction group across all time points (day 1: P = 0.011; day 3: P = 0.013; day 7: P = 0.015), whereas the cortical group only showed a non-significant increasing trend (**Figure 2B**).

Immune checkpoint expression exhibited subset- and location-specific variation (**Figure 2C**). PD-1 expression exhibited distinct temporal dynamics on myeloid subsets between the cortical and subcortical infarction groups. In DCs, PD-1 levels progressively increased in the cortical infarction group but declined in the subcortical infarction group, with significant differences sustained between groups at all observed time points (day 1: P = 0.0233; day 3: P = 0.0098; day 7: P = 0.0184). A comparable pattern was observed in cMono, where PD-1 expression increased in the cortical group but decreased in the subcortical group, again reaching statistical significance at day 3 (P = 0.0193) and day 7 (P = 0.0166). In pDCs, ncMono, and iMono, while no statistically significant differences were reached, visually discernible divergent temporal profiles between the two groups suggest that infarct location may still influence checkpoint regulation dynamics.

PD-L1 expression on iMono demonstrated an early reduction in both groups (days 1 and 3). By day 7, PD-L1 expression increased in the cortical group but continuing to decline in the subcortical group, resulting in a significant difference (P = 0.007). While DCs, ncMono, and cMono followed a comparable non-significant pattern. In contrast, pDCs exhibited an opposite trend, with cortical levels tending to be lower than subcortical, though this did not reach statistically significance.

Overall, the expression levels of CD172a, TIGIT, Tim-3, and VISTA across myeloid subsets did not show significant differences between the cortical and subcortical infarction groups at any of the time points. Detailed expression patterns for these immune checkpoints are presented in **Supplementary Figure 2**.

### 3.4 Dynamic changes in CD4^+^ T cell subsets and immune checkpoints during acute mild ischemic stroke

**Figure 3:**
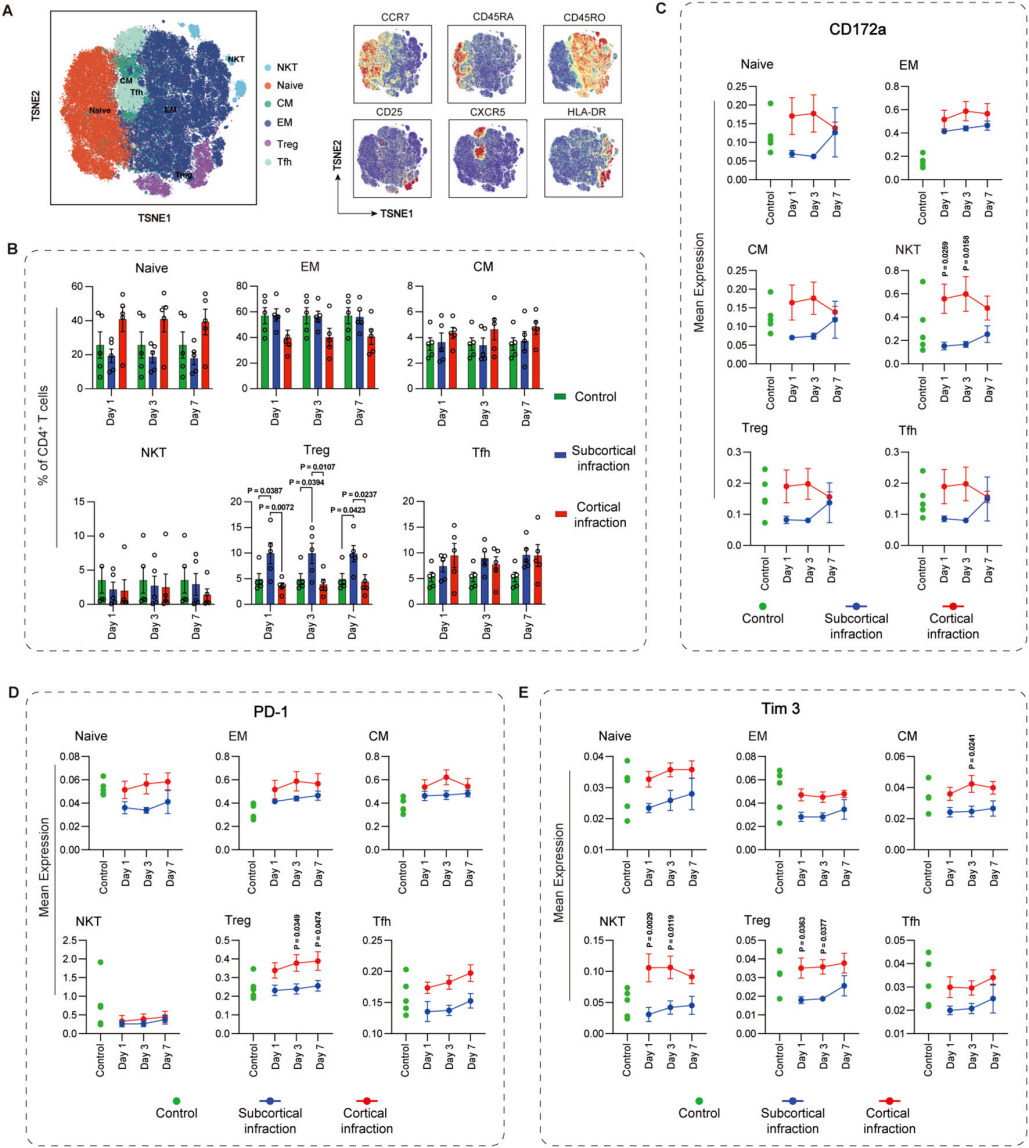
Immune checkpoint expression dynamics across CD4^+^ T cell subsets in cortical and subcortical infarction groups. **(A)** CD4^+^ T cells were annotated into six subsets based on canonical markers: naïve (CD45RA^+^CCR7^+^), central memory (CM; CD45RA^-^CCR7^+^), effector memory (EM; CD45RA^-^CCR7^-^), regulatory T cells (Treg; CD25^+^CD127^-^), follicular helper T cells (Tfh; CXCR5^+^PD-1^+^), and NK-like CD4^+^ T cells (CD56^+^). **(B)** Frequencies of CD4^+^ T-cell subsets across cortical (red) and subcortical (blue) infarction groups. Green bars represent cross-sectionally sampled control subjects and are shown as baseline reference values. **(C–E)** Temporal expression of CD172a, PD-1, and Tim-3 in CD4^+^ T-cell subsets. Green dots represent individual values from the same cross-sectionally sampled control subjects shown in panel B and are presented as baseline reference values. All values are arcsinh-transformed (cofactor = 5). Data represent mean ± SEM Statistical significance is shown as exact P values within the figure.

CD4^+^ T cells were clustered into six functional subsets: naïve, effector memory (EM), central memory (CM), regulatory (Treg), follicular helper (Tfh), and NK-like CD4^+^ T cells (NKT) (**Figure 3A**). Patients with subcortical infarction exhibited significantly higher Treg frequencies than controls across all observed time points (day 1: P = 0.039; day 3: P = 0.039; day 7: P = 0.042). In contrast, Treg levels were consistently reduced in those with cortical infarction compared to the control group (**Figure 3B**). Analysis of checkpoint molecules revealed subset- and time-dependent expression patterns (**Figures 3C–E**). For CD172a, cortical and subcortical infarction groups exhibited opposite temporal trajectories across several CD4^+^ T-cell subsets, including naïve, CM, NKT, Treg, and Tfh cells. In the cortical group, CD172a expression increased during the early phase (days 1 and 3) and declined by day 7, whereas the subcortical group showed a mirrored pattern with early reduction followed by later increase. Notably, the most pronounced divergence occurred in NK-like CD4^+^ T cells, where significant differences were observed at day 1 (P = 0.0259) and day 3 (P = 0.0158). Conversely, in EM CD4^+^ T cells, CD172a expression increased in both groups compared to controls.

PD-1 expression on Treg cells diverged markedly by infarct location. Following cortical infarction, levels increased compared to controls. In contrast, the subcortical group showed no appreciable change from baseline and maintained expression levels consistently below those of the cortical group, leading to significant differences at day 3 (P = 0.0349) and day 7 (P = 0.0474). A similar directional trend— elevated in cortical, reduced in subcortical—were observed in naïve, CM, and Tfh subsets, though without statistical significance.

Tim-3 expression also displayed contrasting trends based on infarct location. In CM T cells, levels were significantly elevated in the cortical group on day 3 (P = 0.0241). Notably, NK-like CD4^+^ T cells exhibited a striking divergence, with Tim-3 increased following cortical infarction but declined after subcortical infarction, resulting in significant group differences at day 1 (P = 0.0029) and day 3 (P = 0.0119). A similar pattern occurred in Tregs, in which Tim-3 expression was markedly decreased in the subcortical group, with significant differences versus the cortical group on days 1 (P = 0.0363) and day 3 (P = 0.0377).

Overall, the expression levels of TIGIT, PD-L1, and VISTA across CD4^+^ T cell subsets did not show significant differences between cortical and subcortical infarction groups at any of the examined time points. Detailed expression patterns for these immune checkpoints are provided in **Supplementary Figure 3**.

### 3.5 Dynamic changes in CD8^+^ T cell subsets and immune checkpoints during acute mild ischemic stroke

**Figure 4:**
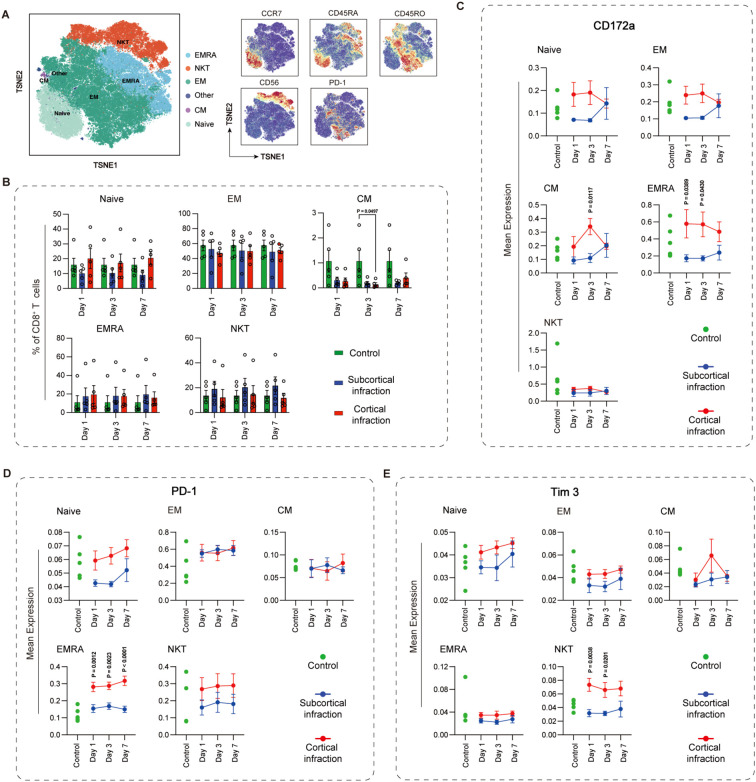
Temporal dynamics and immune checkpoint expression in CD8^+^ T cell subsets in cortical and subcortical infarction groups. **(A)** CD8^+^ T cells were annotated into six subsets: naïve (CD45RA^+^CCR7^+^), CM (CD45RA^-^CCR7^+^), EM (CD45RA^-^CCR7^-^), EMRA (CD45RA^+^CCR7^-^), NK-like CD8^+^ T cells (CD56^+^), and “Other” subsets. **(B)** Frequencies of CD8^+^ T-cell subsets across cortical (red) and subcortical (blue) infarction groups. Green bars represent cross-sectionally sampled control subjects and are shown as baseline reference values. **(C–E)** Temporal expression of CD172a, PD-1, and Tim-3 in CD8^+^ T-cell subsets. Green dots represent individual values from the same cross-sectionally sampled control subjects shown in panel B and are presented as baseline reference values. All values are arcsinh-transformed (cofactor = 5). Data represent mean ± SEM. Statistical significance is shown as exact P values within the figure.

CD8^+^ T cells were classified into six subsets: naïve, effector memory (EM), central memory (CM), effector memory re-expressing CD45RA (EMRA), NK-like CD8^+^ T cells (NKT), and Other (**Figure 4A**). While CM CD8^+^ T cells frequency was significantly reduced in the cortical infarction group at day 3 compared to controls (P = 0.049), with no significant change in the subcortical group (**Figure 4B**). Immune checkpoint profiling revealed several location- and subset-specific alterations (**Figures 4C–E**). CD172a exhibited opposing temporal patterns between the two groups across multiple naïve, EM, CM, and EMRA CD8^+^ T-cells. Among these, the most notable divergence was observed in CM CD8^+^ T cells, where CD172a express Specifically, CD172a expression was significantly higher in the cortical group for CM CD8^+^ T-cells on day 3 (P = 0.0117). Consistent with this, EMRA CD8^+^ T cells also showed significantly higher expression in the cortical group at day 1 (P = 0.0389) and day 3 (P = 0.043). In contrast, NK-like CD8^+^ T cells displayed a downward trend in both groups over time, with no significant intergroup differences.

For PD-1 expression, EMRA and NK-like CD8^+^ T cells showed the most pronounced distinction between infarct locations. PD-1 levels in the cortical group exhibited a progressive increase over all time points, whereas the subcortical group did not show a comparable rise. In EMRA CD8^+^ T cells, this divergence reached statistical significance at every time point (day 1: P = 0.0012; day 3: P = 0.0023; day 7: P <0.0001). A similar, albeit non-significant, directional contrast was observed in naïve CD8^+^ T cells, which displayed a mild decreasing trend in the subcortical group versus an increasing trend in the cortical group.

For Tim-3, NK-like CD8^+^ T cells diverged most strikingly by infarct location, showing increased expression with cortical but decreased expression with subcortical infarction. The significant differences were seen on day 1 (P = 0.0038) and day 3 (P = 0.0201). Naïve CD8^+^ T cells also displayed opposing trajectories between groups. Conversely, in both EM and EMRA CD8^+^ T cells, Tim-3 levels declined over time in the two infarction groups, with no significant intergroup differences.

Overall, the expression levels of TIGIT, PD-L1, and VISTA across CD8^+^ T cell subsets did not show significant differences between cortical and subcortical infarction groups at any of the examined time points. Detailed expression profiles for these immune checkpoints are presented in **Supplementary Figure 4**.

### 3.6 Temporal dynamics and immune checkpoint expression in CCR5^+^ and CCR5^-^ CD8^+^ T cells

**Figure 5:**
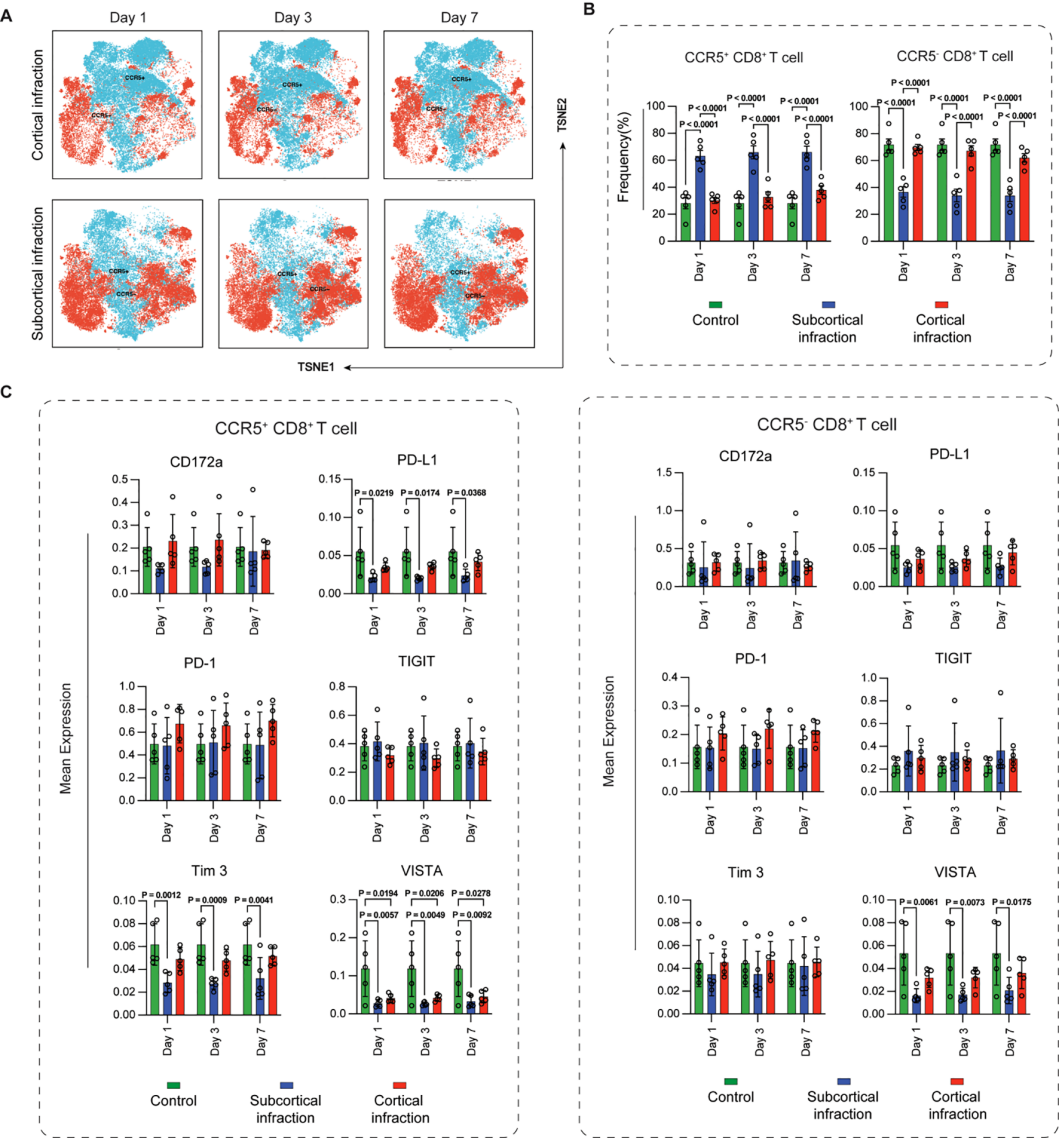
Temporal dynamics and immune checkpoint expression in CCR5^+^ and CCR5^-^ CD8^+^ T cell subsets in cortical and subcortical infarction groups. **(A)** t-SNE visualization of CCR5^+^ and CCR5^-^ CD8^+^ T-cell subsets at days 1, 3, and 7. CCR5 expression was determined using the CCR5 surface marker within clustered CD8^+^ T cells. **(B)** Frequencies of CCR5^+^ and CCR5^-^ subsets in cortical infarction (red) and subcortical infarction (blue). **(C)** Expression of PD-L1, PD-1, CD172a, TIGIT, Tim-3, and VISTA in CCR5^+^ and CCR5^-^ subsets. Green bars represent cross-sectionally sampled control subjects and are shown as baseline reference values. All expression values are arcsinh-transformed (cofactor = 5). Data represent mean ± SEM. Statistical significance is shown as exact P values within the figure.

CCR5-defined CD8^+^ T-cell subsets displayed clear location-dependent distributions (**Figure 5A**). The CCR5^+^ subset was significantly enriched in the subcortical infarction groups compared to control group across all time points (all P < 0.0001). In contrast, no significant difference was observed between the cortical infarction and control groups. Furthermore, a direct comparison between the two patient groups revealed that CCR5^+^ CD8^+^ T cells were significantly more abundant in the subcortical infarction group than in the cortical infarction group across all time points (all P < 0.0001). In contrast, for the CCR5^-^ subset, frequencies were significantly reduced in the subcortical infarction group compared to both the control group and the cortical infarction group at all time points (all P < 0.0001). No significant difference was found between the cortical infarction group and the control group for either subset (**Figure 5B**).

These subsets also displayed distinct checkpoint profiles (**Figure 5C**). Within CCR5^+^ subset, PD-L1 expression was significantly reduced (day 1: P = 0.0219; day 3: P = 0.0174; day 7: P = 0.0368), and Tim-3 expression was consistently lower (day 1: P = 0.0012; day 3: P = 0.0009; day 7: P = 0.0041), in subcortical infarction patients compared with controls across all time points. In CCR5^+^ CD8^+^ T cells, VISTA expression was significantly reduced in both cortical and subcortical infarction patients compared with controls across all time points. The reductions were significant in the cortical group on day 1 (P = 0.0194), day 3 (P = 0.0206), and day 7 (P = 0.0278), and in the subcortical group on day 1 (P = 0.0057), day 3 (P = 0.0049), and day 7 (P = 0.0092). By contrast, in CCR5^-^ CD8^+^ T cells, VISTA expression was significantly decreased only in the subcortical group relative to controls over all time points (day 1: P = 0.0061; day 3: P = 0.0073; day 7: P = 0.0175), whereas no significant differences were observed in the cortical group.

## 4 Discussion

In this study, high-dimensional mass cytometry was employed to delineate peripheral immune dynamics in patients with mild ischemic stroke. Our findings demonstrate that infarct location is a major determinant of systemic immune remodeling. Cortical infarcts were associated with earlier activation of multiple T-cell subsets and more rapid engagement of immune-checkpoint pathways, whereas subcortical infarcts were characterized by sustained monocyte-driven inflammation and delayed but prolonged regulatory adaptations. These patterns underscore the pronounced spatiotemporal heterogeneity of post-stroke immunity and reveal location-specific immune signatures that may have implications for individualized immunomodulatory strategies.

Understanding these differences requires consideration of how distinct neuroanatomical regions interface with the immune system. Cortical and subcortical structures differ substantially in autonomic innervation, blood–brain barrier architecture, and white- versus gray-matter composition (30). Cortical lesions—particularly those involving associative or limbic networks-may induce stronger perturbations of sympathetic–parasympathetic balance, thereby accelerating T-cell activation and checkpoint engagement through splenic and thymic pathways (31). By contrast, subcortical infarcts often disrupt large white-matter tracts and deeper regulatory hubs, promoting sustained release of damage-associated molecular patterns, prolonged monocyte recruitment from the bone marrow and spleen, and delayed initiation of adaptive immune responses (32). These region-dependent neuroimmune interactions offer a mechanistic framework for interpreting the divergent myeloid and lymphoid trajectories observed in this study and are consistent with prior evidence that ischemic stroke topography shapes systemic immunity through both neural and humoral routes.

### 4.1 Influence of infarct location on peripheral immune changes

In this study, high-dimensional mass cytometry allowed us to examine how peripheral immune responses differ between cortical and subcortical infarction in mild ischemic stroke. Across several cell types, the two infarct locations showed distinct directions and time courses of immune changes, rather than simple quantitative differences.

Patients with subcortical infarction showed a more persistent innate inflammatory pattern. Classical monocytes remained elevated from day 1 to day 7, whereas intermediate monocytes were consistently lower. This pattern differs from the expected transition from classical to intermediate subsets during recovery (33, 34). Importantly, previous studies have linked higher intermediate monocyte levels to larger infarct size (35, 36), and lower non-classical monocytes to post-stroke infections (35). Together, these observations suggest that subcortical infarction may create a more sustained inflammatory environment.

In contrast, patients with cortical infarction showed gradual increases in regulatory or memory-related subsets across both lymphoid and myeloid lineages. Immune checkpoint molecules on T cells and dendritic cells also appeared earlier in cortical infarcts, indicating faster initiation of regulatory pathways.

Several biological differences between cortical and subcortical regions may contribute to these patterns. Cortical areas are closely connected with autonomic and limbic networks, which can rapidly influence peripheral immune organs. Subcortical regions contain large white-matter tracts and deeper structural hubs; injury in these areas may lead to continued release of damage-associated signals, supporting prolonged monocyte recruitment (37, 38). These mechanisms could explain the opposite tendencies observed in classical monocytes, dendritic cells, and selected T-cell subsets.

Overall, our findings support the concept that infarct location meaningfully shapes peripheral immune responses after mild ischemic stroke. Understanding these location-specific immune features may help refine immunomodulatory strategies in clinical practice, particularly with respect to the timing and targeting of innate versus adaptive immune pathways.

### 4.2 Subcortical infarction sustains a myeloid-dominant inflammatory response

Myeloid populations exhibited clear and sustained differences between cortical and subcortical infarction. In patients with subcortical infarction, classical monocytes remained significantly elevated from day 1 through day 7, whereas cortical infarction patients showed only a mild and non-significant increase relative to controls. In parallel, intermediate monocytes were consistently reduced after subcortical infarction, while the cortical group demonstrated a modest upward trend over time. This pattern contrasts with the typical post-stroke trajectory, in which classical monocytes predominate early and gradually transition toward intermediate and non-classical subsets during recovery (33, 34). Previous studies have reported associations between higher intermediate monocyte levels and larger infarct volumes (35, 36), as well as links between reduced non-classical monocytes and increased susceptibility to post-stroke infections (35). Collectively, the sustained elevation of classical monocytes combined with a blunted recovery of intermediate monocytes suggests that subcortical infarction is associated with a more persistent systemic inflammatory state.

Immune checkpoint profiling of myeloid subsets further supports this interpretation. PD-1 expression on dendritic cells and classical monocytes increased over time in cortical infarction patients but declined after subcortical infarction, with significant differences observed at most examined time points. Although the functional implications of PD-1 expression on myeloid cells require cautious interpretation (39–41), the opposing temporal patterns observed here are consistent with the proposed role of PD-1 signaling in restraining antigen-presenting cell activation. PD-L1 expression on intermediate monocytes also diverged between groups: both cortical and subcortical infarction showed early reductions after ischemic stroke, but by day 7 the cortical group demonstrated partial recovery, whereas PD-L1 levels in subcortical infarction patients declined further. These findings reflect the context-dependent roles of the PD-1/PD-L1 axis, in which PD-1 signaling may protect tissues by limiting excessive immune activation (42, 43), while excessive or sustained PD-L1 expression has been implicated in post-stroke systemic immunosuppression (19). Consistent with this notion, experimental studies have shown that enhancing PD-L1 expression in monocytes can promote functional recovery in animal models of ischemic stroke (44).

Taken together, these results indicate that subcortical infarction is characterized by a more prolonged and less tightly regulated myeloid-driven inflammatory response, marked by persistent elevation of classical monocytes and weaker engagement of immune checkpoint pathways. In contrast, cortical infarction appears to initiate regulatory signaling earlier, which may help constrain excessive inflammation. These distinctions may contribute to differences in systemic immune vulnerability across ischemic stroke subtypes and further underscore infarct location as a biologically meaningful determinant of post-stroke immune responses.

### 4.3 Cortical infarcts preferentially reshape adaptive immune regulation

Beyond the myeloid compartment, infarct location was also associated with distinct patterns of adaptive immune engagement. The most consistent difference between groups was observed in Treg dynamics, which increased early after subcortical infarction but remained comparatively lower in cortical infarction. Because Treg expansion has been linked to inflammatory cues and autonomic associated signaling pathways (45, 46), this divergence is consistent with differences in neuroimmune input between cortical and subcortical regions. Cortical ischemic strokes—particularly those involving associative or limbic circuits—may be associated with less pronounced autonomic perturbation during the acute phase, potentially delaying the systemic signals required for early Treg expansion. In contrast, subcortical lesions often affect deeper structures and white-matter tracts that are more closely connected to autonomic regulatory pathways, which may favor earlier induction of regulatory programs.

Checkpoint expression patterns across CD4^+^ T-cell subsets provided additional evidence for location-specific immune shaping. CD172a, PD-1, and Tim-3 displayed opposing temporal trajectories across naïve, central memory, Tfh, Treg and NK-like CD4^+^ T cells populations in cortical versus subcortical infarction. In cortical infarction, early increases in PD-1 and CD172a were consistent with more rapid initiation of adaptive regulatory signaling. Given the established roles of PD-1 in restraining excessive T-cell activation and promoting tolerance (47), and of CD47–SIRPα signaling in coordinating innate-adaptive immune interactions (48), such early upregulation may help counterbalance acute inflammatory activation. By contrast, subcortical infarction exhibited delayed or inverse changes, consistent with a more sustained inflammatory milieu that is slower to recruit inhibitory programs.

Tim-3 expression revealed one of the clearest distinctions between groups. In NK-like CD4^+^ T cells and Treg cells, cortical infarction was associated with early increases in Tim-3, whereas subcortical infarction showed early reductions. Because Tim-3 functions as an inhibitory receptor limiting effector activity and cytokine production (49, 50), this divergence further supports the notion that cortical infarcts engage adaptive immune restraint earlier, while subcortical infarcts maintain a more permissive inflammatory environment during the acute phase.

Parallel patterns were observed in CD8^+^ T-cell subsets. PD-1 expression increased progressively in EMRA and NK-like CD8^+^ T cells following cortical infarction, whereas subcortical infarction showed minimal change. This distinction is notable given that EMRA CD8^+^ T cells possess strong cytotoxic potential, which has been implicated in exacerbating tissue injury when insufficiently regulated. The increase in PD-1 after cortical infarction may therefore contribute to limiting excessive cytotoxic activity, while the blunted response in subcortical infarction could leave these effector populations less constrained. Similar group-specific differences in Tim-3 expression further point to earlier engagement of adaptive immune regulation in cortical infarction.

Taken together, these findings suggest that cortical infarction is associated with earlier activation of adaptive immune regulatory responses, whereas subcortical infarction shows delayed or weaker regulatory engagement, allowing inflammatory activity to persist. Differences in immune checkpoint expression across CD4^+^ and CD8^+^ T-cell subsets further highlight that infarct location influences the timing and strength of immune regulation after ischemic stroke. In this setting, variation in regulatory signaling is likely to affect not only T-cell activation but also their ability to migrate and adopt distinct functional states. Therefore, whether infarct location further shapes T-cell trafficking and effector behavior through chemokine-related pathways warrants closer examination.

### 4.4 CCR5 defines functionally distinct CD8^+^ T cell states after ischemic stroke

CCR5 stratification revealed a clear functional division among CD8^+^ T cells across infarct locations. CCR5^+^ CD8^+^ T cells were consistently enriched in patients with subcortical infarction at all examined time points, whereas cortical infarction did not show comparable increases. In contrast, CCR5^-^ CD8^+^ T cells were relatively more abundant in cortical infarction, suggesting that infarct location influences the balance between trafficking-prone effector populations and more regulated CD8^+^ T-cell states.

These findings are consistent with prior evidence that CCR5 promotes T-cell recruitment to inflamed tissues and contributes to inflammatory injury in experimental models of ischemic stroke (51, 52). Subcortical infarction, characterized by a more intense and prolonged myeloid-driven inflammatory milieu, may therefore favor the induction of CCR5 ligands and enhance effector T-cell trafficking. Indeed, the canonical CCR5 ligands CCL3, CCL4, and CCL5 are known to be upregulated in white-matter injury and can amplify local and systemic chemotactic signaling (53), providing a plausible mechanistic link between infarct topology and CCR5^+^ CD8^+^ T-cell accumulation.

Immune checkpoint expression further distinguished CCR5-defined CD8^+^ T-cell subsets. Within CCR5^+^ CD8^+^ T cells, PD-L1 and Tim-3 expression was consistently reduced in subcortical infarction, indicating a more activated and less tightly regulated effector phenotype. VISTA expression was markedly reduced in CCR5^+^ subsets in both cortical and subcortical infarction, suggesting a broader suppression of this inhibitory pathway following ischemic stroke. In contrast, within CCR5^-^ CD8^+^ T cells, VISTA expression was selectively reduced in subcortical infarction but relatively preserved in cortical infarction, again highlighting infarct location as a determinant of inhibitory signaling strength within distinct CD8^+^ T-cell compartments.

Together, these results identify CCR5 as a marker of functionally divergent CD8^+^ T-cell states that are differentially shaped by cortical and subcortical infarction. Subcortical infarction preferentially promotes the accumulation of CCR5^+^ effector CD8^+^ T cells with weaker engagement of immune checkpoint pathways, whereas cortical infarction maintains a higher proportion of CCR5^-^ CD8^+^ T cells with relatively preserved inhibitory regulation. These distinctions suggest that CCR5 may serve not only as a biomarker of post-stroke immune activation but also as a potential therapeutic target whose relevance may depend on infarct location and the underlying immune landscape (54).

## 5 Limitations

Several limitations should be acknowledged. First, the study was restricted to patients with mild ischemic stroke, which limits the generalizability of our findings to moderate or severe cases in which systemic inflammation and immune dysregulation tend to be more pronounced. Second, the relatively small sample size reduced statistical power, particularly for low-frequency immune subsets, and may have contributed to variability in group comparisons. Third, the analysis focused on the acute phase (days 1, 3, and 7); although these timepoints capture well-defined phases of early post-stroke immune activation and modulation, the absence of later timepoints precludes evaluation of longer-term immune trajectories and their relationship to recovery. Fourth, infarct size may influence peripheral immune responses; although large territorial infarctions were not included, the lack of quantitative infarct-volume measurements limits our ability to fully separate the impact of infarct size from that of infarct location. Fifth, although cortical and subcortical infarctions were defined using standard MRI criteria, mixed-pattern lesions may not be fully captured by binary classification.

Finally, the CyTOF antibody panel was designed primarily to evaluate immune-checkpoint pathways and therefore did not include several trafficking or functional markers-such as CXCR3, CCR6, and key NK-cell activation receptors-which constrained the resolution of specific T-cell and NK-cell subpopulations. In addition, CyTOF provides relative rather than absolute cell frequencies, which may limit direct comparison with conventional hematology parameters. Moreover, controls were sampled in a cross-sectional manner and served as baseline references rather than longitudinal comparators, which precludes direct assessment of temporal immune dynamics in non-stroke individuals. Future studies with larger cohorts, a broader severity spectrum, voxel-based neuroimaging, extended panel design, and longer-term follow-up will be essential to delineate the full spatiotemporal complexity of post-stroke immune remodeling.

## 6 Conclusion

This study demonstrates that infarct location is a key determinant of peripheral immune remodeling in mild ischemic stroke. Subcortical infarction sustained a myeloid-dominant inflammatory program, marked by persistently elevated classical monocytes and delayed checkpoint engagement, whereas cortical infarction preferentially reshaped adaptive immune regulation, with earlier modulation of CD4^+^ and CD8^+^ T-cell subsets and distinct checkpoint signaling patterns. In addition, the differential distribution of CCR5^+^ and CCR5^-^ CD8^+^ T cells highlight a location-dependent diversification of effector phenotypes.

Together, these findings reveal that post-stroke immunity is not uniform but strongly shaped by lesion topology, providing mechanistic insight into the spatiotemporal heterogeneity of immune responses after ischemic stroke. Recognition of these infarct-specific immune signatures may help refine patient stratification and guide the development of more targeted immunomodulatory strategies in the future.

## Data Availability

The data presented in the study are deposited in the Mendeley Data repository, accession number DOI: 10.17632/bpmrbntb3p.1.
