# Supplementary material for: CyTOF profiling identifies location-specific peripheral immune checkpoint and immune cell subset in mild ischemic stroke
Source: Front Immunol. 2026 Jan 27;17:1739324. doi: 10.3389/fimmu.2026.1739324 (PMC12886039; doi:10.3389/fimmu.2026.1739324)
Supplement: Supplementary file 1 [file DataSheet1.docx]

**Supplementary figure legends:**

**Figure S1. Study design and workflow.** (A) Timeline of peripheral blood collection from ischemic stroke patients classified into cortical or subcortical infarction groups. Samples were obtained at 24 h, 72 h, and 7 d after onset. (B) Workflow of mass cytometry (CyTOF) analysis. PBMCs were isolated, stained with a 42-marker antibody panel, and acquired on a Helios CyTOF instrument. Raw FCS files were debarcoded, normalized using EQ™ calibration beads, drift-corrected, batch-aligned, and arcsinh-transformed (cofactor = 5). Live, single, CD45⁺ cells were obtained after excluding beads (140Ce⁻), debris (191Ir⁺/193Ir⁺), dead cells (194Pt⁻), and doublets (event length < 20). Downstream analyses included X-shift clustering, t-SNE visualization, and subset-level quantification.

**Figure S2. Longitudinal expression of CD172a, TIGIT, Tim-3 and VISTA in Myeloid cell subsets following mild ischemic stroke with cortical or subcortical infarction.**
Myeloid cells were clustered and annotated using canonical markers into classical monocytes (CD14⁺CD16⁻), intermediate monocytes (CD14⁺CD16⁺), non-classical monocytes (CD14⁻CD16⁺), cDCs (CD11c⁺HLA-DR⁺), and pDCs (CD123⁺HLA-DR⁺). The expression of CD172a, TIGIT, Tim-3 and VISTA was quantified using arcsinh-transformed signal intensities. Peripheral blood was collected from patients with cortical (red) or subcortical infarction (blue) on days 1, 3, and 7 after symptom onset. Green dots represent individual values from control subjects sampled in a cross-sectional manner. Controls were not longitudinally followed and are shown as baseline reference values rather than as a temporal trajectory.Data are presented as mean ± SEM (n = 5 per group).

**Figure S3. Longitudinal expression of TIGIT, PD-L1 and VISTA in CD4⁺ T cell subsets following mild ischemic stroke with cortical or subcortical infarction.**
CD4⁺ T cells were gated as singlet, live lymphocytes and subsequently subdivided into six functional T-cell subsets based on canonical markers: naïve CD8⁺ T cells (CD3⁺CD8⁺CD45RA⁺CCR7⁺), effector memory (EM) CD8⁺ T cells (CD3⁺CD8⁺CD45RA⁻CCR7⁻), central memory (CM) CD8⁺ T cells (CD3⁺CD8⁺CD45RA⁻CCR7⁺), natural killer T (NKT) cells (CD3⁺CD56⁺), regulatory T cells (Treg; CD4⁺CD25⁺FoxP3⁺), and T follicular helper (Tfh) cells (CD4⁺CXCR5⁺PD-1⁺). The expression of TIGIT, PD-L1 and VISTA was quantified using arcsinh-transformed signal intensities. Peripheral blood was collected from patients with cortical (red) or subcortical infarction (blue) on days 1, 3, and 7 after symptom onset. Green dots represent individual values from control subjects sampled in a cross-sectional manner. Controls were not longitudinally followed and are shown as baseline reference values rather than as a temporal trajectory.Data are presented as mean ± SEM (n = 5 per group).

**Figure S4. Temporal expression of TIGIT, PD-L1 and VISTA in CD8⁺ T cell subsets following mild ischemic stroke with cortical or subcortical infarction.**CD8⁺ T cells were gated from CD45⁺ live lymphocytes and classified into five subsets according to differentiation markers: naïve (CD45RA⁺CCR7⁺), central memory (CM; CD45RA⁻CCR7⁺), effector memory (EM; CD45RA⁻CCR7⁻), terminally differentiated EMRA cells (CD45RA⁺CCR7⁻), and natural killer T–like CD8⁺ cells (CD8⁺CD56⁺). The expression of TIGIT, PD-L1 and VISTA was assessed using arcsinh-transformed intensities. Peripheral blood samples were obtained from patients with cortical (red) or subcortical infarction (blue) on days 1, 3, and 7 post-stroke. Green dots represent individual values from control subjects sampled in a cross-sectional manner. Controls were not longitudinally followed and are shown as baseline reference values rather than as a temporal trajectory.Data are shown as mean ± SEM (n = 5 per group).
