# Supplementary material for: CyTOF profiling identifies location-specific peripheral immune checkpoint and immune cell subset in mild ischemic stroke
Source: Front Immunol. 2026 Jan 27;17:1739324. doi: 10.3389/fimmu.2026.1739324 (PMC12886039; doi:10.3389/fimmu.2026.1739324)
Supplement: Supplementary file 2 [file DataSheet2.docx]

**Supplementary Tables**

**Supplementary T****able 1. Antibody panel used for mass cytometry analysis**

**
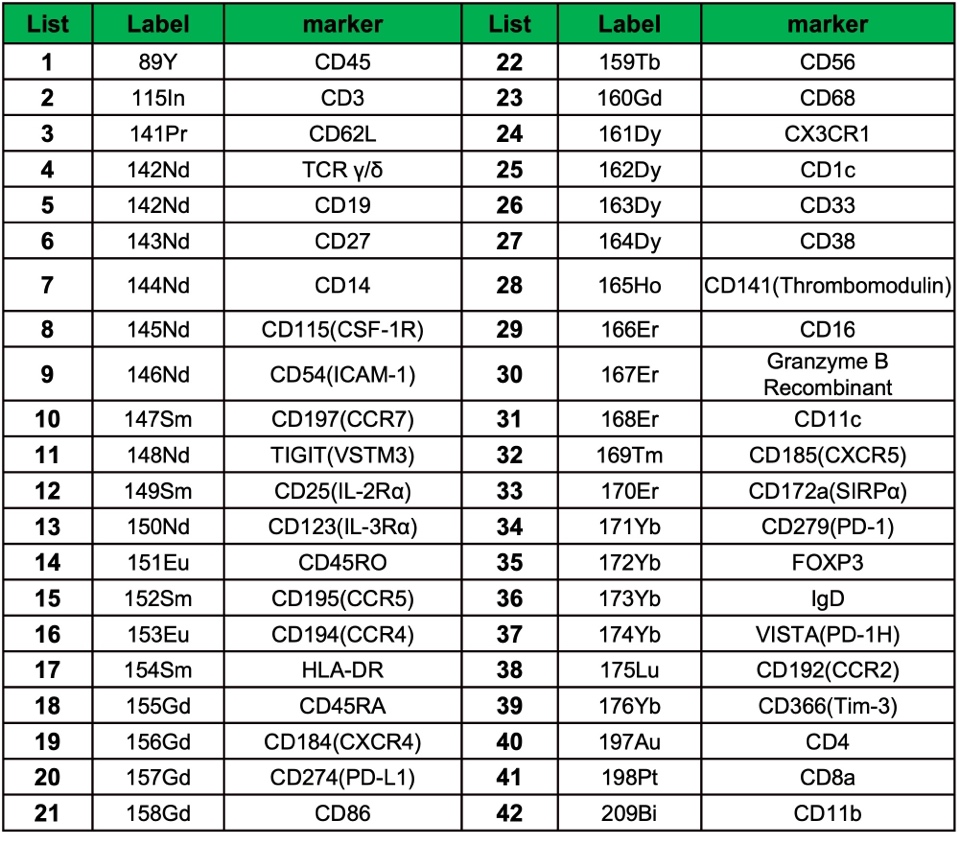
**

**Supplementary Table 2. Marker definitions for major immune lineages and sub-clusters**

| **A.Major immune lineages(11 clusters)** | |
| --- | --- |
| Population | Marker Definition |
| B cells | CD45⁺CD19⁺ |
| CD4⁺T cells: | CD45⁺CD3⁺CD4⁺ |
| CD8⁺T cells | CD45⁺CD3⁺CD8⁺ |
| γδT cells | CD45⁺CD3⁺TCRγδ⁺ |
| NK cells | CD45⁺CD3⁻CD56⁺ |
| NKT cells | CD45⁺CD3⁺CD56⁺ |
| Monocytes | CD45⁺CD14/CD16 variable expression |
| cDCs | CD45⁺HLA-DR⁺CD11c⁺CD33⁺ |
| pDCs | CD45⁺CD123⁺HLA-DR^dim |
| Basophils | CD45⁺CD123⁺CD11b⁻HLA-DR⁻ |
| Undefined | Did not meet lineage thresholds |
| **B.Myeloid sub-clusters** | |
| Subset | Marker Definition |
| Classical monocytes(cMono) | CD14⁺CD16⁻ |
| Intermediate monocytes(iMono) | CD14⁺CD16⁺ |
| Non-classical monocytes(ncMono) | CD14⁻CD16⁺ |
| cDCs | HLA-DR⁺CD11c⁺CD33⁺ |
| pDCs | CD123⁺HLA-DR^dim |
| **C.CD4⁺T-cell sub-clusters** | |
| Subset | Marker Definition |
| Naïve CD4⁺T | CD45RA⁺CCR7⁺ |
| Central memory(CM) | CD45RO⁺CCR7⁺ |
| Effector memory(EM) | CD45RO⁺CCR7⁻ |
| T follicular helper(Tfh) | CXCR5⁺ |
| NK-like CD4⁺T | CD56⁺ |
| **D.CD8⁺T-cell sub-clusters** | |
| Subset | Marker Definition |
| Naïve CD8⁺T | CD45RA⁺CCR7⁺ |
| Central memory(CM) | CD45RO⁺CCR7⁺ |
| Effector memory(EM) | CD45RO⁺CCR7⁻ |
| EMRA | CD45RA⁺CCR7⁻ |
| NK-like CD8⁺T | CD56⁺ |
| Other CD8⁺T | Not meeting above definitions |
| **E.CCR5-defined CD8⁺T-cell states** | |
| Subset | Marker Definition |
| CCR5⁺CD8⁺T | CD3⁺CD8⁺CCR5⁺ |
| CCR5⁻CD8⁺T | CD3⁺CD8⁺CCR5⁻ |
