# Supplementary figures and images for: CyTOF profiling identifies location-specific peripheral immune checkpoint and immune cell subset in mild ischemic stroke
Source: Front Immunol. 2026 Jan 27;17:1739324. doi: 10.3389/fimmu.2026.1739324 (PMC12886039; doi:10.3389/fimmu.2026.1739324)

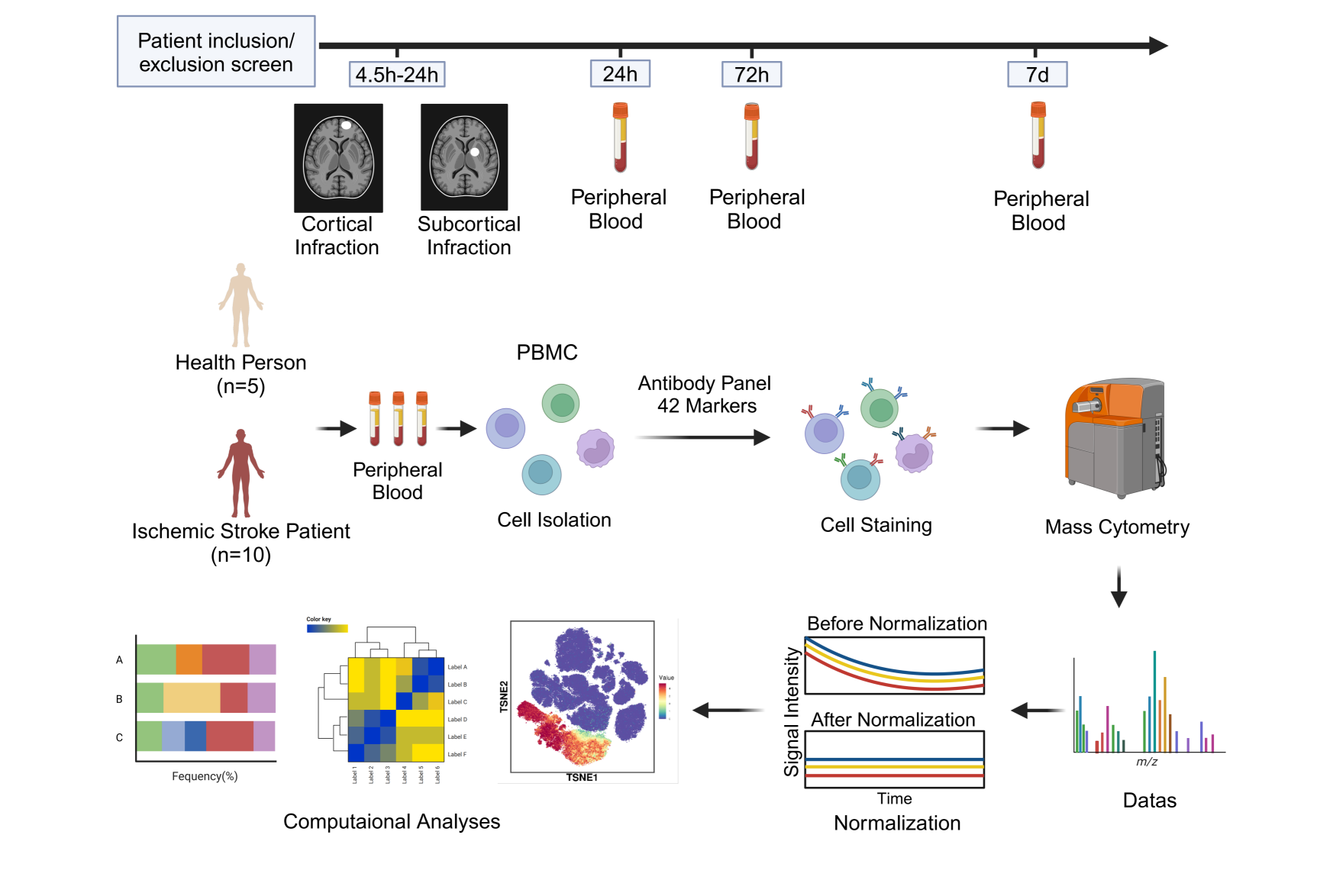

Supplement: Supplementary file 3 [file Image1.tiff]

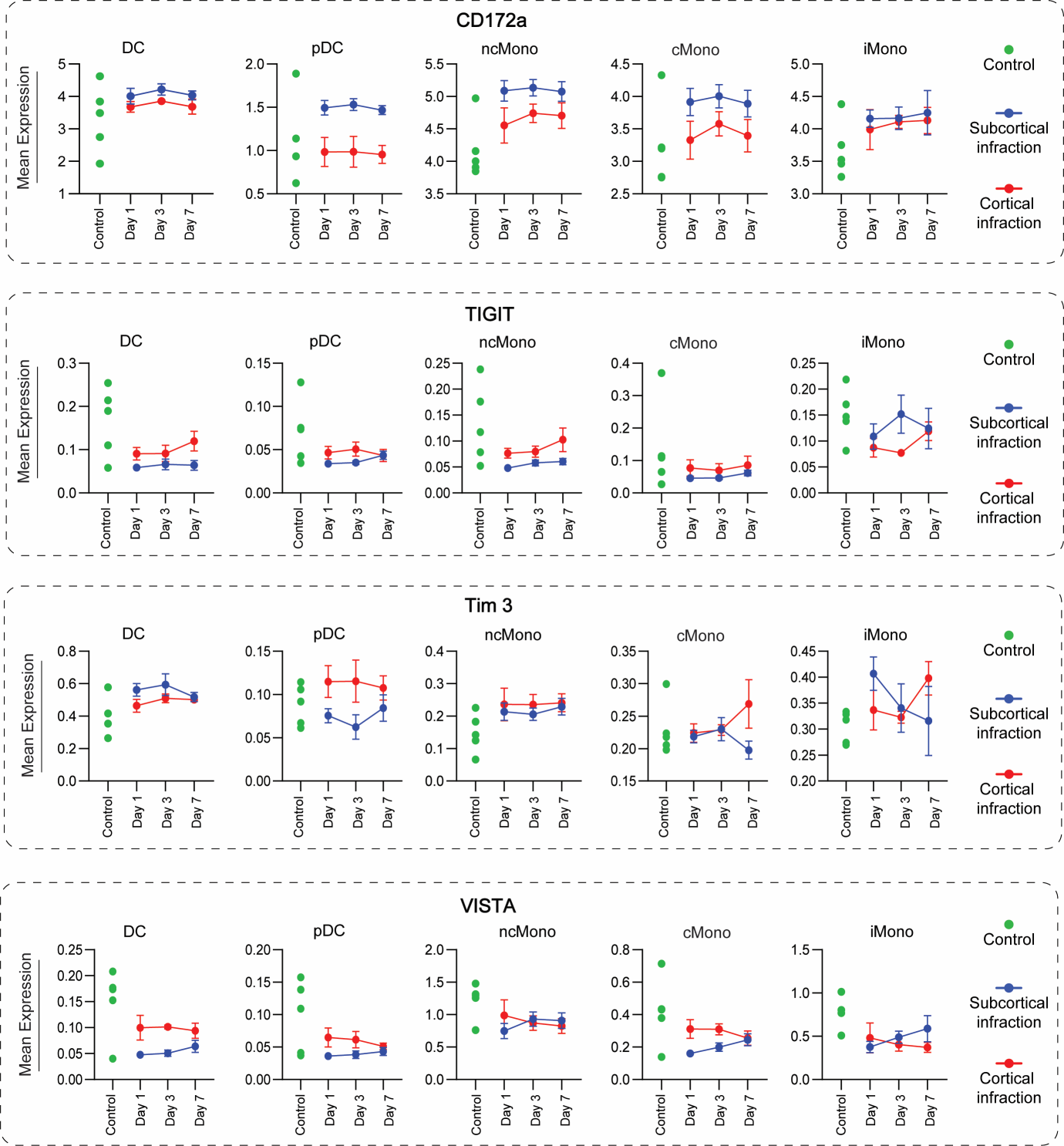

Supplement: Supplementary file 4 [file Image2.tiff]

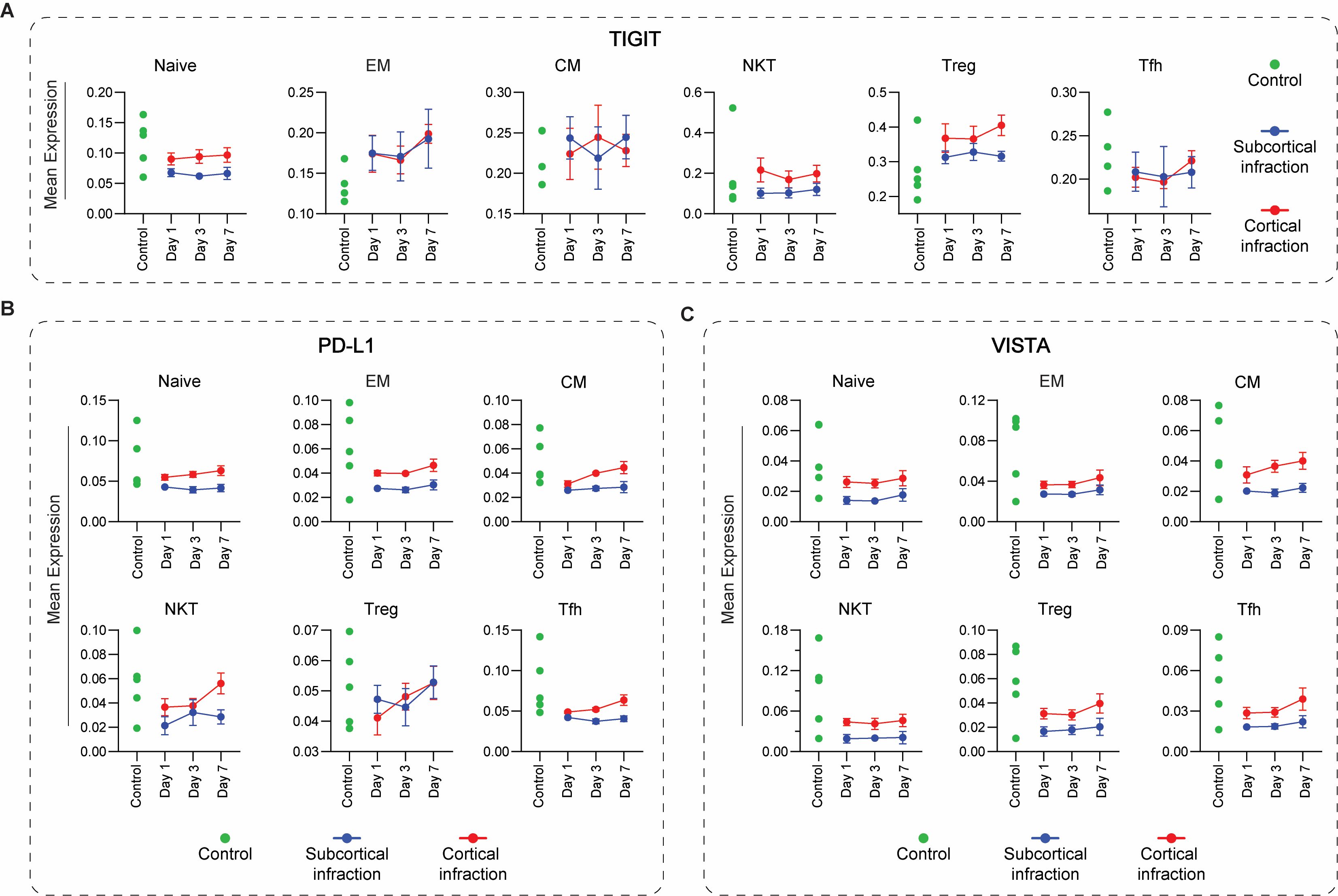

Supplement: Supplementary file 5 [file Image3.tiff]

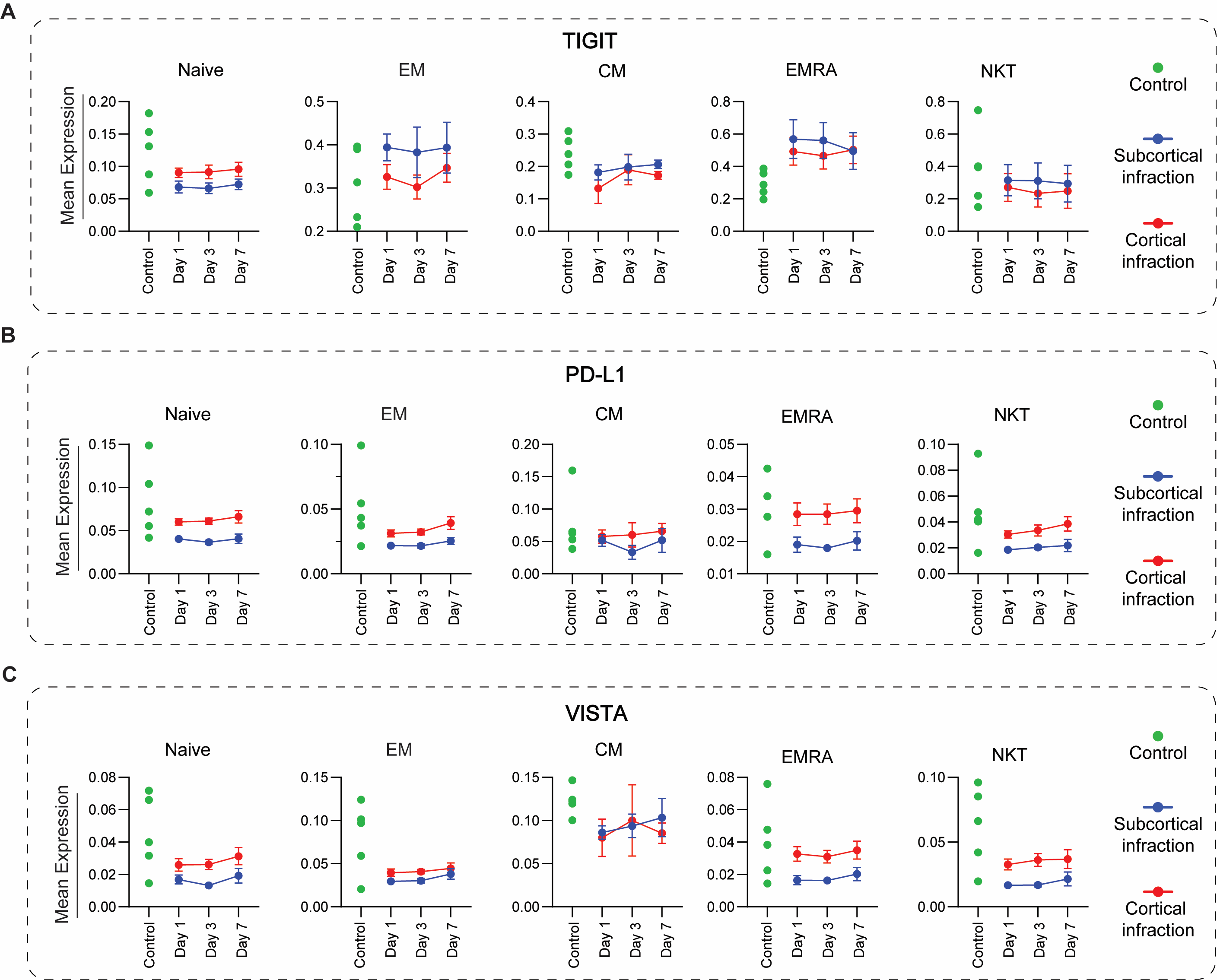

Supplement: Supplementary file 6 [file Image4.tiff]
